# Nonlinear associations between maternal depressive symptoms and children’s mental health: a cross-sectional study

**DOI:** 10.1186/s40359-026-04007-5

**Published:** 2026-01-19

**Authors:** Fenling Feng, Xin Zhang, Jihua Hu

**Affiliations:** 1 Department of pediatrics, Xi’an International Medical Center Hospital, Xi’an, Shaanxi China; 2 Department of pediatrics, Tianyou Children’s Hospital of Chang’an, Xi’an, Shaanxi China; 3 Department of Pharmacy, Xi’an Jiaotong University School of Medicine Affiliated Honghui Hospital, Xi’an, Shaanxi China; 4 Center for Clinical Skills Training, Xi’an Children’s Hospital (Affiliated to Xi’an Jiaotong University), Xi’an, Shaanxi China; 5 Center for Clinical Skills Training, Xi’an Children’s Hospital (Affiliated Children’s Hospital of Xi’an Jiaotong University, 69 Xi Ju Road, Xi’an, 710003 Shaanxi China

**Keywords:** Maternal depressive symptoms, Preschool children, CES‑D, SDQ, Cross‑sectional study

## Abstract

**Background:**

While associations between maternal depressive symptoms (MDS) and offspring mental health have been documented, the precise nature of this relationship, particularly potential nonlinear patterns, remains insufficiently characterized.

**Objective:**

This study sought to examine potential nonlinear dose-response relationships between MDS and child mental health among preschool-aged children.

**Methods:**

We conducted a cross-sectional investigation enrolling 17,115 mother-child dyads from kindergartens in a city in western China. MDS were assessed using the Center for Epidemiologic Studies Depression Scale (CES-D). Children’s mental health was assessed via maternal reports using the Strengths and Difficulties Questionnaire (SDQ). Data were collected between February 28 and March 5, 2025.

**Results:**

Among the 17,115 mother-child dyads, 16.14% (*n* = 2,763) of mothers (mean age: 34.49 ± 4.65 years) reported elevated depressive symptoms (CES-D score ≥ 16). A nonlinear dose-response relationship was observed, with turning points at maternal CES-D scores of 17 for total difficulties and internalizing problems, 21 for externalizing problems, and 14 for prosocial behavior problems. Below these thresholds, stronger associations were observed (OR = 1.20 for total difficulties, OR = 1.16 for internalizing problems, OR = 1.10 for externalizing problems, and OR = 1.11 for prosocial behavior problems), while above the thresholds, the associations were substantially weakened.

**Conclusion:**

This cross-sectional study identified distinctive nonlinear dose-response associations between MDS and children’s mental health outcomes in a western Chinese city. The associations were stronger below specific thresholds while weakening above these thresholds. These findings warrant further investigation and validation.

**Supplementary Information:**

The online version contains supplementary material available at 10.1186/s40359-026-04007-5.

## Introduction

Children’s mental health problems have profound implications for individual development and society. Research indicates that psychological issues, including internalizing problems (such as anxiety and depression), externalizing problems (such as impulsivity and attention-deficit/hyperactivity disorders) [1, 2], and deficits in prosocial behavior [3], often exhibit high stability from early childhood through adolescence and into adulthood [4–6]. These problems not only affect children’s current quality of life but may also lead to decreased academic performance, difficulties in social adaptation, and increased risk of mental illness in adulthood [7].

Maternal Depressive Symptoms (MDS) are widely recognized as one of the key factors influencing children’s psychological development [8–10], with substantial evidence demonstrating significant associations with various mental health outcomes [11]. Multiple theoretical mechanisms explain these associations, operating across biological, interpersonal, and family-system levels. From a biological perspective, children of depressed mothers may inherit genetic vulnerabilities that increase susceptibility to psychological difficulties [12]. From a relational perspective, depressed mothers often exhibit reduced emotional responsiveness, less positive engagement, and inconsistent discipline strategies, which can disrupt children’s emotional regulation development [13, 14]. At the family-system level, MDS can contribute to broader disruptions in family functioning, including increased family conflict, reduced social support, and compromised family cohesion [15]. These mechanisms are not mutually exclusive but rather operate interactively and cumulatively across child development, and different dimensions of child mental health (e.g., internalizing vs. externalizing problems, prosocial behavior) may show differential sensitivity to MDS through these distinct pathways [10, 16].

While numerous studies indicate significant associations between maternal depression and various child psychological problems [1], other investigations have reported weak or non-significant relationships in specific contexts. For instance, Pelham et al. [17] employing propensity score matching to control for potential confounding factors, found that the apparent association between MDS and child behavioral problems was substantially attenuated. Similarly, Han et al. [18] found that MDS were not directly related to childhood depression. These inconsistent findings may be partially explained by the heterogeneity in maternal depression trajectories and their differential impacts on child outcomes. Longitudinal studies have demonstrated that MDS exhibit distinct trajectory patterns (e.g., low-stable, high-declining, moderate-increasing) across the early parenting period [19]. Importantly, different trajectory patterns are associated with varying levels of child psychopathology [19]. This pattern of heterogeneous trajectories has been further corroborated by a longitudinal cohort study identifying different maternal depression trajectory groups (e.g., no symptoms, mild symptoms, moderate symptoms) from pregnancy through the postpartum period, which show differential associations with child behavioral problems at different developmental stages [20]. The heterogeneity in longitudinal depression trajectories, wherein different severity levels and temporal patterns yield differential child outcomes, suggests that the relationship between MDS and child mental health may not be uniformly linear across the full range of symptom severity. Specifically, the differential associations observed across trajectory groups indicate that the strength of association between MDS and child outcomes may vary systematically at different levels of symptom severity. If such variation exists, it would imply potential non-linear patterns in the relationship between MDS and child mental health. However, whether this complexity, which has been observed longitudinally across trajectory groups, also manifests as detectable non-linear patterns in cross-sectional assessments, and how such patterns may differ across various dimensions of child mental health, remains empirically underexplored. Examining potential non-linear associations in a cross-sectional framework may thus provide an alternative methodological perspective to explore the dose-response characteristics of the relationship between MDS and child mental health.

We hypothesize that there is a potentially non-linear association between MDS and child mental health. This study aimed to investigate the potential non-linear relationships between MDS and preschool children’s mental health outcomes in a sample of mothers and children from a city in western China.

## Method

### Study population

This cross-sectional study was conducted between February 28 and March 5, 2025, in collaboration with the Municipal Education Bureau and local kindergartens. Children aged 3–6 years and their mothers were recruited from 189 public kindergartens across all 13 districts/counties in the city, Shaanxi Province, China. Inclusion criteria were: (1) children aged 3–6 years enrolled in the selected kindergartens; (2) mothers who completed the questionnaire on both their own depressive symptoms and their child’s emotional and behavioral problems; and (3) complete data on core assessment measures including CES-D and SDQ. Exclusion criteria included: (1) children with previously diagnosed severe developmental disorders or physical disabilities that would preclude valid assessment of emotional and behavioral problems; and (2) questionnaires with missing data on the core assessment measures.

The sampling process, designed and implemented by the research team, utilized a stratified cluster approach based on administrative regions. Public kindergartens were randomly selected within each stratum using government-registered lists provided by the Education Bureau. Enrollment targets were proportionally allocated according to each district’s preschool-aged population to ensure demographic representativeness.

Data collection was coordinated by trained research staff in collaboration with kindergarten teachers. All questionnaires were completed voluntarily with informed consent. The final population consisted of 17,115 mother-child dyads. Missing data were observed for maternal age (*n* = 340) and child age (*n* = 525). After excluding missing variables, the total sample size was 16,258 mother-child dyads.

## Measures

### Children’s mental health

The Strengths and Difficulties Questionnaire (SDQ) [21–23] was completed by mothers. The SDQ evaluates total difficulties score (TDS), internalizing problems, externalizing problems, and prosocial behavior. The SDQ comprises 25 items rated on a 3-point scale (0–2), yielding five subscales: emotional symptoms, conduct problems, hyperactivity/inattention, peer relationship problems, and prosocial behavior. The TDS was calculated by summing the first four subscales (excluding prosocial behavior). It should be noted that the prosocial behavior subscale measures a positive strength, with higher scores indicating better prosocial skills, whereas higher scores on the other four subscales indicate greater difficulties.

Based on validated Chinese cut-off values, children were categorized as at-risk if they scored > 14 for TDS, > 3 for emotional symptoms, > 2 for conduct problems, > 6 for hyperactivity/inattention, > 4 for peer problems, and < 6 for prosocial behavior problems [21, 22]. Specifically, a score below 6 on the prosocial behavior subscale indicates prosocial behavior problems. Children were dichotomously categorized (Yes/No) for internalizing problems based on the presence of emotional symptoms, and for externalizing problems based on conduct problems or hyperactivity/inattention [21, 23].

This categorization approach follows the methodology validated in large-scale epidemiological studies with Chinese children aged 3–6 years [21, 22], ensuring age-appropriate and culturally validated thresholds. Our choice of dichotomization approach was based on: (1) validated Chinese normative data specific to preschool-aged children; and (2) consistency with prior epidemiological research in similar populations. Different SDQ dimension integration methods and cut-off values exist across studies, which may affect cross-study comparisons.

The SDQ was used with official permission from Youthinmind Limited for academic research purposes, as authorized for our research team.

### Maternal depressive symptoms

The Center for Epidemiologic Studies Depression Scale (CES-D) [24] was employed to assess MDS. This validated self-report instrument comprises 20 items evaluating depressive symptom frequency during the preceding week. Each item utilizes a four-point response scale ranging from 0 (none) to 3 (all of the time), yielding total scores between 0 and 60. A CES-D score of ≥ 16 was used as the threshold to indicate elevated depressive symptoms [17]. This cutoff has been validated in studies of maternal depression, where total scores of 16 or greater indicate significant probability of depressive disorder [17]. And at-risk groups could be identified through the recommended cutoff values. The utility of this threshold has been further supported by systematic reviews across diverse populations [24].

### Covariates

Demographic and socioeconomic variables were assessed at the time of data collection. Maternal age and child age were recorded in years at the time of data collection. Child gender was categorized as boy or girl. Maternal education level was assessed based on the highest level of education completed and categorized as below high School, high School, above high School. Maternal employment status was classified as employed or unemployed. Maternal marital status was categorized as married or non-married (including divorced, separated, widowed, or never married). Annual family income, in thousands of ¥, was self-reported and categorized as < 100, or ≥ 100 [21].

### Statistical analysis

In the statistical analysis, continuous variables were described as means ± standard deviation, whereas categorical variables were expressed as counts and percentages. Maternal CES-D scores were processed both as continuous variables and as categorical variables for analysis. In primary analyses, SDQ outcomes were dichotomized. While dichotomization may result in information loss, sensitivity analyses using continuous SDQ scores were conducted to assess robustness of findings.

To investigate correlations between variables and child mental health outcomes, we conducted univariate logistic regression analyses. For unadjusted logistic regression, maternal age was categorized into three groups reflecting reproductive age ranges, and child age into three groups reflecting key developmental stages in the preschool period. Generalized additive model (GAM) was employed to investigate the dose-response relationship between MDS and children’s mental health. We then employed a two-piecewise linear regression model to examine the threshold effect of MDS on children’s mental health. The turning point for maternal CES-D scores was identified through exploratory analysis, where potential turning points were systematically tested within a pre-defined interval, and the one that maximized the likelihood function was selected as the optimal threshold [25]. Bootstrap resampling method generated the 95% CI for the observed turning point [25], following the methodology described in previous studies [26, 27], ensuring robust estimation of the threshold effect. To evaluate the nonlinearity of the association, logarithm likelihood ratio test was employed to compare the two-piecewise linear model with the conventional linear model. This test evaluates whether the two-piecewise linear model provides a significantly better fit to the data than the one-line linear regression model.

To assess robustness of our findings, two sensitivity analyses were conducted. First, to address missing data (maternal age: 1.98%; child age: 3.07%), dummy variables indicating missingness were included as covariates in all models (*N* = 17,115). Dummy variables were created to indicate missing covariate values for continuous variables with missingness exceeding 1% [25]. Consistency with primary analyses (complete cases: *N* = 16,258) was evaluated by comparing statistical significance, threshold values with 95% CIs, and effect sizes. Second, to account for potential kindergarten-level clustering, Generalized Additive Mixed Model (GAMM) [28] with kindergarten as a random intercept was fitted. Clustering effects were evaluated using the intraclass correlation coefficient (ICC) and design effect (DE) [29], with values near 0 and 1, respectively, indicating negligible clustering. All models included smooth terms for MDS and adjusted for all covariates.

All key measures in this study were based on maternal reports, which may introduce common method bias. To assess this concern, Harman’s single-factor test [30] was conducted. All 20 items from the CES-D scale and 25 items from the SDQ (45 items total) were simultaneously entered into an exploratory factor analysis. The appropriateness of the data for factor analysis was first examined using the Kaiser-Meyer-Olkin (KMO) measure of sampling adequacy and Bartlett’s test of sphericity. Principal component analysis was then employed to extract unrotated factors. Following established methodological recommendations, common method bias was considered substantial if the first unrotated factor accounted for more than 50% of the total variance [30].

Significance was defined as a 2-sided *P* < 0.05. Statistical processing of all data was performed with EmpowerStats (X&Y Solutions, Inc., Boston, MA; www.empowerstats.com) and R version 4.2.0.

## Results

### Characteristics of the study population

Table 1 demonstrates that among 17,115 mother-child pairs, 16.14% (*n* = 2,763) of mothers reported elevated depressive symptoms. Regarding demographic characteristics, mothers with elevated depressive symptoms were slightly older than those with lower scores (34.49 ± 4.65 vs. 34.30 ± 4.38 years, *P* = 0.046), though this difference was modest in practical terms. Children of mothers with elevated depressive symptoms were also slightly older (4.91 ± 0.86 vs. 4.81 ± 0.89 years, *P* < 0.001). No significant difference was observed in child gender distribution between the two groups (51.43% vs. 50.77% boys, *P* = 0.528), indicating that maternal depressive symptom levels were not associated with child sex in this sample. Mothers with elevated depressive symptoms differed significantly from those with lower scores across multiple sociodemographic characteristics: they had lower education levels (above high school: 22.48% vs. 28.63%, *P* < 0.001), higher unemployment rates (42.38% vs. 31.60%, *P* < 0.001), higher proportions of non-married status (4.52% vs. 1.94%, *P* < 0.001), and lower proportions of high-income families (10.57% vs. 19.18%, *P* < 0.001). Unlike the group of mothers with lower depressive symptom scores, children whose mothers reported depressive symptoms exhibited higher proportions of TDS (38.33% vs. 13.92%), internalizing problems (38.87% vs. 16.59%), externalizing problems (38.22% vs. 21.17%), and prosocial behavior problems (59.54% vs. 46.11%) (all *P* < 0.001).

**Table 1:** Characteristics of the study population (*N* = 17,115)

Characteristics	Maternal CES-D score		*P*-value
	<16 (*n* = 14352)	≥ 16 (*n* = 2763)	
Maternal age (y)	34.30 ± 4.38	34.49 ± 4.65	0.046
Child age (y)	4.81 ± 0.89	4.91 ± 0.86	< 0.001
Maternal CES-D score	9.05 ± 3.89	22.51 ± 6.78	< 0.001
Child gender			0.528
Boy	7287 (50.77%)	1421 (51.43%)	
Girl	7065 (49.23%)	1342 (48.57%)	
Maternal education level			< 0.001
Below high School	3831 (26.69%)	1045 (37.82%)	
High School	6412 (44.68%)	1097 (39.70%)	
Above high School	4109 (28.63%)	621 (22.48%)	
Maternal employment status			< 0.001
Employed	9817 (68.40%)	1592 (57.62%)	
Unemployed	4535 (31.60%)	1171 (42.38%)	
Annual family income, in thousands, ¥			< 0.001
<¥100	11,599 (80.82%)	2471 (89.43%)	
≥¥100	2753 (19.18%)	292 (10.57%)	
Maternal marital status			< 0.001
Married	14,073 (98.06%)	2638 (95.48%)	
Non-married	279 (1.94%)	125 (4.52%)	
Total difficulties score			< 0.001
No	12,354 (86.08%)	1704 (61.67%)	
Yes	1998 (13.92%)	1059 (38.33%)	
Internalizing problems			< 0.001
No	11,971 (83.41%)	1689 (61.13%)	
Yes	2381 (16.59%)	1074 (38.87%)	
Externalizing problems			< 0.001
No	11,313 (78.83%)	1707 (61.78%)	
Yes	3039 (21.17%)	1056 (38.22%)	
Prosocial behavior problems			< 0.001
No	7735 (53.89%)	1118 (40.46%)	
Yes	6617 (46.11%)	1645 (59.54%)	

Data are expressed as mean ± SD or number (percentage)

¥, yuan renminbi (to convert to US dollar, multiply by 7.30)

Among the 17,115 mother-child dyads, the amount of missing values for the covariates were 340 (1.98%) for maternal age, 525 (3.07%) for child age

### Common method bias assessment

Given the reliance on maternal reports for all measures, potential common method bias was assessed using Harman’s single-factor test. The analysis demonstrated excellent data suitability for factor analysis, with a KMO value of 0.956 and a significant Bartlett’s test of sphericity (χ² = 366,656.470, df = 990, *P* < 0.001). The first unrotated factor accounted for 24.68% of the total variance, substantially below the 50% threshold criterion. These results suggest that common method bias did not substantially influence the study findings.

### Unadjusted association between variables and children’s mental health

Table 2 shows the univariate logistic models. Compared with mothers with CES-D scores < 16, those with elevated depressive symptoms had significantly higher odds of having children with total difficulties (OR = 3.84, 95% CI 3.51–4.20, *P* < 0.0001), internalizing problems (OR = 3.20, 95% CI 2.93–3.49, *P* < 0.0001), externalizing problems (OR = 2.30, 95% CI 2.11–2.51, *P* < 0.0001), and prosocial behavior problems (OR = 1.72, 95% CI 1.58–1.87, *P* < 0.0001). The prevalence of TDS problems, internalizing problems, externalizing problems, and prosocial behavior problems in the total sample was 17.87% (3,057/17,115), 20.19% (3,455/17,115), 23.93% (4,095/17,115), and 48.27% (8,262/17,115), respectively (see Supplementary Table S1-S4). Higher maternal education and annual family income were associated with lower odds of child mental health problems, with mothers having above high school education showing reduced odds for total difficulties (OR = 0.44, 95% CI 0.39–0.49, *P* < 0.0001) and higher family income associated with lower odds across all domains (OR = 0.56–0.83, all *P* < 0.0001). Conversely, maternal unemployment was associated with higher odds of total difficulties (OR = 1.46, 95% CI 1.34–1.58, *P* < 0.0001) and internalizing problems (OR = 1.54, 95% CI 1.42–1.66, *P* < 0.0001).

**Table 2:** Unadjusted association between variables and children’s mental health

		Total difficulties score	Internalizing problems	Externalizing problems	Prosocial behavior problems
	Statistics	OR (95% CI) *P* value	OR (95% CI) *P* value	OR (95% CI) *P* value	OR (95% CI) *P* value
Maternal CES-D Score	11.22 ± 6.68	1.11 (1.10, 1.11) < 0.0001	1.09 (1.09, 1.10) < 0.0001	1.07 (1.06, 1.07) < 0.0001	1.05 (1.05, 1.06) < 0.0001
Maternal CES-D Score					
<16	14,352 (83.86%)	Reference	Reference	Reference	Reference
≥16	2763 (16.14%)	3.84 (3.51, 4.20) < 0.0001	3.20 (2.93, 3.49) < 0.0001	2.30 (2.11, 2.51) < 0.0001	1.72 (1.58, 1.87) < 0.0001
Maternal age (y)					
≤ 30	2613 (15.58%)	Reference	Reference	Reference	Reference
> 30 ≤ 40	12,571 (74.94%)	0.72 (0.65, 0.80) < 0.0001	0.80 (0.73, 0.89) < 0.0001	0.87 (0.79, 0.96) 0.0064	0.90 (0.82, 0.97) 0.0104
>40	1591 (9.48%)	0.68 (0.58, 0.80) < 0.0001	0.68 (0.58, 0.79) < 0.0001	0.80 (0.69, 0.93) 0.0028	0.69 (0.60, 0.78) < 0.0001
Child age (y)					
≤ 4	3892 (23.46%)	Reference	Reference	Reference	Reference
> 4 ≤5	5495 (33.12%)	0.92 (0.82, 1.02) 0.1134	1.07 (0.97, 1.19) 0.1955	0.86 (0.78, 0.94) 0.0016	0.72 (0.66, 0.78) < 0.0001
> 5	7203 (43.42%)	0.92 (0.84, 1.02) 0.1299	1.09 (0.99, 1.21) 0.0763	0.82 (0.75, 0.90) < 0.0001	0.61 (0.57, 0.66) < 0.0001
Child gender					
Boy	8708 (50.88%)	Reference	Reference	Reference	Reference
Girl	8407 (49.12%)	0.92 (0.85, 1.00) 0.0434	1.12 (1.04, 1.21) 0.0030	0.91 (0.84, 0.97) 0.0061	0.82 (0.78, 0.87) < 0.0001
Maternal education level					
Below high School	4876 (28.49%)	Reference	Reference	Reference	Reference
High School	7509 (43.87%)	0.60 (0.55, 0.66) < 0.0001	0.65 (0.59, 0.70) < 0.0001	0.85 (0.78, 0.92) 0.0001	0.84 (0.78, 0.91) < 0.0001
Above high School	4730 (27.64%)	0.44 (0.39, 0.49) < 0.0001	0.49 (0.44, 0.54) < 0.0001	0.84 (0.76, 0.92) 0.0002	0.75 (0.69, 0.81) < 0.0001
Maternal employment status					
Employed	11,409 (66.66%)	Reference	Reference	Reference	Reference
Unemployed	5706 (33.34%)	1.46 (1.34, 1.58) < 0.0001	1.54 (1.42, 1.66) < 0.0001	1.04 (0.97, 1.12) 0.2744	1.12 (1.06, 1.20) 0.0003
Annual family income, in thousands, ¥					
<¥100	14,070 (82.21%)	Reference	Reference	Reference	Reference
≥¥100	3045 (17.79%)	0.56 (0.50, 0.63) < 0.0001	0.58 (0.52, 0.64) < 0.0001	0.83 (0.75, 0.91) < 0.0001	0.75 (0.69, 0.81) < 0.0001
Maternal marital status					
Married	16,711 (97.64%)	Reference	Reference	Reference	Reference
Non-married	404 (2.36%)	1.70 (1.36, 2.13) < 0.0001	1.67 (1.35, 2.08) < 0.0001	1.18 (0.94, 1.47) 0.1458	0.82 (0.67, 1.00) 0.0556

¥, yuan renminbi (to convert to US dollar, multiply by 7.30)

### Identification of nonlinear association

**Fig. 1:**
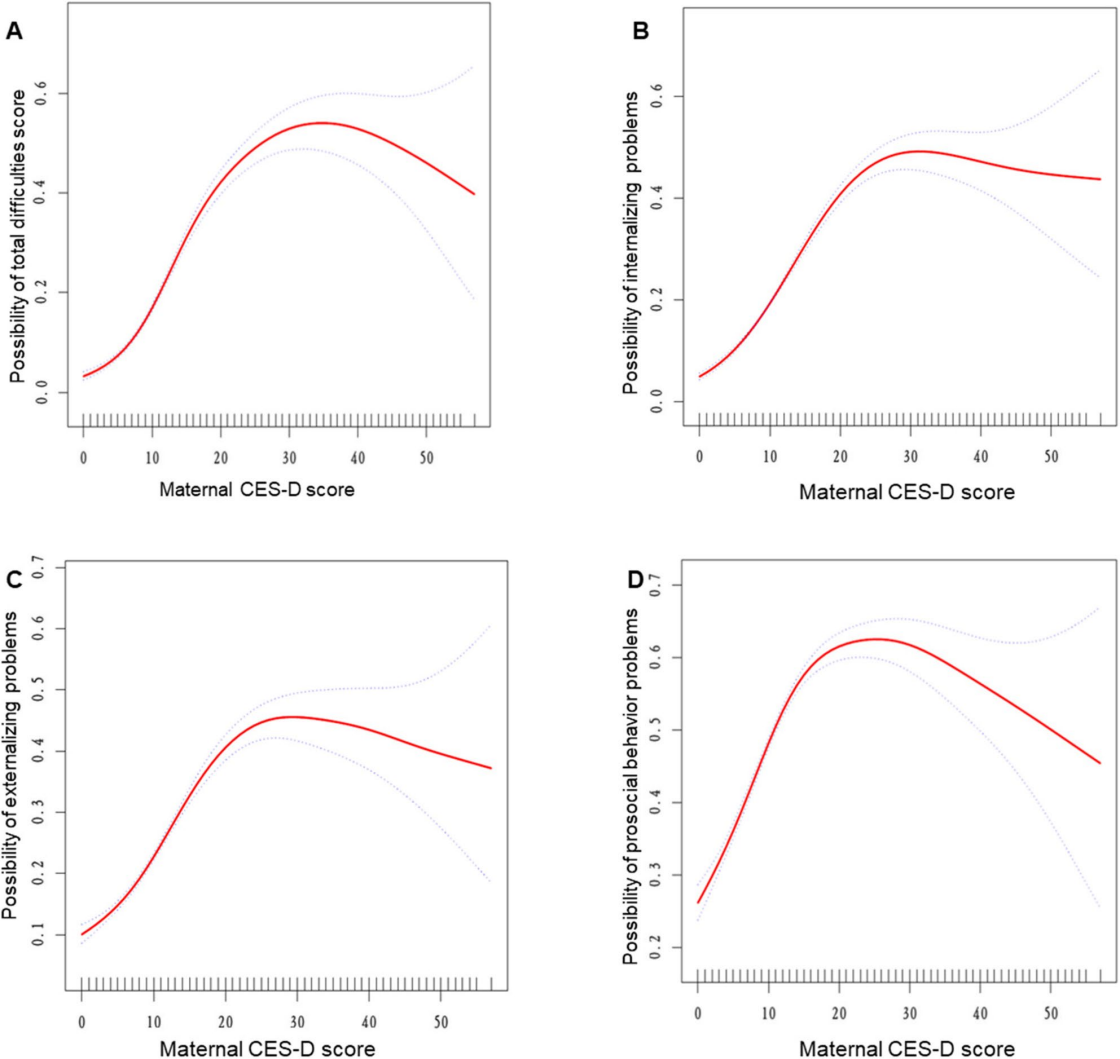
Associations between MDS and children’s mental health. **A** MDS and TDS; **B** MDS and internalizing problems; **C** MDS and externalizing problems; **D** MDS and prosocial behavior problems. Solid red line represents the smooth curve fit between variables. Blue bands represent the 95% of confidence interval from the fit

Using a generalized additive model, we identified a threshold, nonlinear relationship between MDS and child mental health outcomes (Fig. 1). The relationship is illustrated by a solid red line representing the fitted smooth curve, with blue bands indicating the 95% confidence intervals. The model was adjusted for potential confounding factors, including maternal characteristics (age, education level, employment status, and marital status), child characteristics (age and gender), and annual family income level.

The one-line linear regression model and a two-piecewise linear model were compared, and the *P* value of the log-likelihood ratio test was < 0.001. A *P* value < 0.001 indicates that the two-piecewise linear model fits the data significantly better, providing statistical justification for the presence of a threshold effect at the inflection point. The turning points were identified at maternal CES-D scores of 17 (95% CI 15–18) for both total difficulties and internalizing problems, 21 (95% CI 16–23) for externalizing problems, and 14 (95% CI 13–16) for prosocial behavior problems. (Table 3). Below these thresholds, the associations between MDS and children’s mental health problems were stronger, whereas above these thresholds, the associations were substantially attenuated.

**Table 3:** Threshold effect analysis of the maternal depressive symptoms and children’s mental health (*N* = 16,258)

	Total difficulties score	Internalizing problems	Externalizing problems	Prosocial behavior problems
	OR (95%CI) *P* value	OR (95%CI) *P* value	OR (95%CI) *P* value	OR (95%CI) *P* value
Model I				
One line effect	1.10 (1.10, 1.11) < 0.0001	1.09 (1.08, 1.09) < 0.0001	1.07 (1.06, 1.07) < 0.0001	0.95 (0.94, 0.95) < 0.0001
Model II				
Turning point (K)	17	17	21	14
CES-D < K	1.20 (1.18, 1.21) < 0.0001	1.16 (1.14, 1.17) < 0.0001	1.10 (1.09, 1.11) < 0.0001	1.11 (1.10, 1.12) < 0.0001
CES-D > K	1.03 (1.01, 1.04) < 0.0001	1.02 (1.01, 1.03) 0.0007	1.00 (0.98, 1.01) 0.2958	1.00 (1.00, 1.01) 0.3218
*P* value for LRT test*	< 0.001	< 0.001	< 0.001	< 0.001
95% CI for turning point	15, 18	15, 18	16, 23	13, 16

Data were presented as OR (95% CI) *P* value; Model I, linear analysis; Model II, non-linear analysis. Adjusted for maternal age (years), child age (years), child gender, maternal education level, maternal employment status, maternal marital status, and annual family income level

*Abbreviations*: *CI* confidence interval, *OR* odds ratio, *LRT* logarithm likelihood ratio test

*P < 0.05 indicates that model II is significantly different from Model I

### Sensitivity analyses

Missing data sensitivity analyses (*N* = 17,115) showed high consistency with primary analyses (*N* = 16,258). All associations remained significant (all *P* < 0.001), and threshold values were identical or nearly identical: 17 for total difficulties (both analyses), 17 for internalizing problems (both analyses), 21 for externalizing problems (both analyses), and 14 vs. 15 for prosocial behavior problems (overlapping 95% CIs: 13–16 vs. 14–17). Effect sizes showed minimal variation, with ORs differing by < 1% across all outcomes (Supplementary Table S5).

GAMM analyses revealed negligible kindergarten-level clustering (ICC ≈ 0.000, DE = 1.00 for all outcomes; Supplementary Table S6). Smooth terms for MDS remained highly significant (Supplementary Figure S1), with nonlinear patterns consistent with primary GAM findings.

Sensitivity analyses using continuous SDQ scores (Supplementary Table S7 and Figure S2) addressed potential information loss and demonstrated threshold consistency with primary findings. All continuous analyses confirmed stronger associations below thresholds and attenuated effects above, supporting the robustness of nonlinear relationships.

## Discussion

This cross-sectional study investigated the association between MDS and offspring mental health in a large sample of mother-child pairs from a city in western China. To our knowledge, this study appears to be the first attempt to quantitatively analyze the nonlinear associations between MDS and specific dimensions of children’s mental health, providing novel insights into how these associations vary across different severity levels of MDS.

An important finding of this study is the non-linear association pattern between MDS and child psychopathology. Our findings indicate that the strength of associations varies across different dimensions of children’s mental health, with distinct statistical thresholds at which the associations become significant. For example, MDS below these thresholds were associated with increased difficulties across multiple behavioral domains in children, alongside prosocial behavior problems (indicating reduced social adaptability). It is important to note that, given the cross-sectional nature of our data, these patterns reflect statistical associations rather than causal or temporal relationships.

Our results align with previous research demonstrating the associations between MDS and child mental health problems. Prior studies have consistently reported that maternal depression is associated with higher levels of internalizing and externalizing problems in children, as well as lower prosocial behavior [31–33]. By exploring specific inflection points, our study extends the existing literature and underscores the need for more nuanced analyses of MDS and their associations with children’s mental health.

The nonlinear threshold patterns identified in this study offer important theoretical contributions to understanding associations between MDS and child mental health. While existing research has consistently documented associations between MDS and child outcomes [1, 13], few studies have systematically examined whether these associations follow nonlinear patterns or exhibit threshold effects. Our findings extend this literature by demonstrating that association strengths vary systematically across severity ranges, being strongest within specific ranges and attenuating beyond identified thresholds. From a risk accumulation perspective, our results suggest that associations may exhibit threshold-dependent variations rather than increasing monotonically across the full severity range [34]. Specifically, our results demonstrate varying thresholds across different mental health domains, further suggesting potential differential associations, with certain aspects (e.g., prosocial behavior) showing stronger associations at lower symptom levels while others (e.g., externalizing problems) show stronger associations at higher levels. This domain-specific pattern is consistent with risk accumulation models [34] and differential susceptibility theory [35]. These findings highlight the importance of considering nonlinear patterns and domain-specific variations in research on maternal depression and child mental health.

Notably, the present study found that associations between MDS and children’s mental health problems weakened or became non-significant beyond the identified threshold turning points. Several speculative explanations may account for this pattern. One possibility is ceiling effects in the outcome measures, where limited score ranges may constrain the observable variation at extreme levels of MDS. When children’s problem scores cluster near the upper bounds of the assessment scales, the tools may have reduced sensitivity to detect incremental associations. Alternatively, this pattern might reflect unmeasured moderating factors (e.g., paternal involvement, social support) [36] that become more influential at higher levels of maternal depression, potentially buffering the associations. The varying thresholds across different mental health domains suggest potential differential sensitivities to MDS severity [10], though this interpretation remains tentative. These threshold patterns suggest that MDS below these thresholds show stronger associations with child mental health problems, suggesting that mothers with mild-to-moderate depressive symptoms may warrant particular clinical attention. However, given the cross-sectional nature of this study, these interpretations should be considered preliminary and require validation through longitudinal research and mechanistic research.

The turning point for externalizing problems was notably higher than that for internalizing problems, a difference that reflects differential sensitivity and developmental mechanisms across different types of mental health problems in response to MDS. Existing research demonstrates that MDS have a stronger impact on children’s internalizing problems than on externalizing problems [1]. In contrast, the development of externalizing problems involves more complex multi-factorial interactions and stronger moderating mechanisms. Particularly, children’s stress reactivity as a physiological-psychological trait can serve as a protective factor that buffers the adverse effects of maternal depression on children’s externalizing behavioral problems [37]. These protective factors enable children to maintain relatively low levels of externalizing behavioral problems when facing low-to-moderate levels of MDS; only when MDS reach higher levels can they overcome the buffering effects of these protective factors, leading children to exhibit significant externalizing behavioral problems.

The lower threshold for prosocial behavior compared to other outcomes may reflect the unique nature of prosocial behavior as a dimension of children’s mental health. Prosocial behavior, which encompasses actions such as sharing, helping, and showing empathy, is closely tied to the quality of mother-child interactions [38]. Research has shown that current maternal depression is associated with worsened children’s social outcomes, including compromised prosocial behavior and peer relationships [39]. Maternal depression impairs maternal sensitive-responsive parenting – the mother’s ability to notice and correctly interpret her child’s signals or needs, and provide contingent, appropriate, and consistent responses to these signals or needs – which serves as a key mediator between maternal depression and child mental health outcomes [40]. Even subtle disruptions in maternal emotional availability or responsiveness may disproportionately affect the development of prosocial skills, particularly during early childhood when these behaviors are being established [41, 42].

The observed thresholds have important implications for understanding the association between MDS and children’s mental health. Our finding suggests that elevated MDS are associated with greater difficulties in child mental health outcomes.

### Limitations

Several important considerations should be emphasized when interpreting our findings. To our knowledge, this is the first study to quantitatively examine non-linear associations and threshold patterns between MDS and child mental health outcomes. Given the novelty and complexity of this research question, our findings should be regarded as preliminary and interpreted with appropriate caution. These results require further validation to confirm their robustness.

Additionally, several methodological limitations should be acknowledged. First, as an observational study, unmeasured confounding factors may exist despite our adjustment for multiple demographic and socioeconomic characteristics. A further limitation includes missing data for variables. Second, the cross-sectional design precludes causal inference regarding the relationship between MDS and child mental health. Third, all measures were based on maternal self-reports. Although Harman’s single-factor test suggested limited impact of common method bias, common source variance remains a potential concern. Finally, our stratified cluster sampling approach focused on public kindergartens with children aged 3–6 years in a city in western China, limiting the generalizability of findings to private kindergartens, other age brackets, or different locations. Future research with larger samples is needed to validate the threshold effects, elucidate the underlying mechanisms, and examine these associations across diverse populations and developmental stages.

## Conclusion

In conclusion, our cross-sectional analysis of 17,115 mother-child dyads from a western Chinese city revealed significant nonlinear associations between MDS and children’s mental health outcomes. We identified distinctive threshold effects where associations demonstrated varying strength at different symptom levels, highlighting the complexity of maternal-child mental health interactions beyond conventional linear assumptions. These findings suggest that attention to MDS – even at subclinical levels – may be important for child mental health clinical assessment. Future research is needed to examine whether these threshold effects are consistent across diverse populations and cultural contexts.

## Supplementary Information 

Supplementary Material 1

## Data Availability

The datasets generated and analyzed during this study are available from the corresponding author on reasonable request.

## Abbreviations

MDS: Maternal depressive symptoms
TDS: Total difficulties score
