# Supplementary material for: Nonlinear associations between maternal depressive symptoms and children’s mental health: a cross-sectional study
Source: BMC Psychol. 2026 Jan 19;14:219. doi: 10.1186/s40359-026-04007-5 (PMC12896353; doi:10.1186/s40359-026-04007-5)
Supplement: Supplementary file 1 — Supplementary Material 1 [file 40359_2026_4007_MOESM1_ESM.docx]

**Table S1. Supplementary data for Table 2**

|  | **Total difficulties score** | |  |
| --- | --- | --- | --- |
|  | No (n=14058) | Yes (n=3057) | *P*-value |
| **Maternal CES-D Score** | 10.32 ± 6.12 | 15.37 ± 7.53 | <0.001 |
| **Maternal CES-D Score** |  |  | <0.001 |
| <16 | 12354 (87.88%) | 1998 (65.36%) |  |
| ≥16 | 1704 (12.12%) | 1059 (34.64%) |  |
| **Maternal age (y)** |  |  | <0.001 |
| ≤30 | 2038 (14.75%) | 575 (19.45%) |  |
| >30 ≤40 | 10447 (75.60%) | 2124 (71.85%) |  |
| >40 | 1334 (9.65%) | 257 (8.69%) |  |
| **Child age (y)** |  |  | 0.223 |
| ≤4 | 3169 (23.20%) | 723 (24.68%) |  |
| >4 ≤5 | 4544 (33.26%) | 951 (32.47%) |  |
| >5 | 5948 (43.54%) | 1255 (42.85%) |  |
| **Child Gender** |  |  | 0.043 |
| Boy | 7102 (50.52%) | 1606 (52.54%) |  |
| Girl | 6956 (49.48%) | 1451 (47.46%) |  |
| **Maternal educational level** |  |  | <0.001 |
| Below high School | 3665 (26.07%) | 1211 (39.61%) |  |
| High School | 6263 (44.55%) | 1246 (40.76%) |  |
| Above high School | 4130 (29.38%) | 600 (19.63%) |  |
| **Maternal employment status** |  |  | <0.001 |
| employed | 9588 (68.20%) | 1821 (59.57%) |  |
| unemployed | 4470 (31.80%) | 1236 (40.43%) |  |
| **Annual family income, in thousands, ¥** |  |  | <0.001 |
| <¥100 | 11371 (80.89%) | 2699 (88.29%) |  |
| ≥¥100 | 2687 (19.11%) | 358 (11.71%) |  |
| **Maternal marital status** |  |  | <0.001 |
| Married | 13762 (97.89%) | 2949 (96.47%) |  |
| non-married | 296 (2.11%) | 108 (3.53%) |  |

Data are expressed as number (percentage).

Note: ¥, yuan renminbi (to convert to US dollar, multiply by 7.30).

**Table S2. Supplementary data for Table 2**

|  | **Internalizing problems** | |  |
| --- | --- | --- | --- |
|  | No (n=13660) | Yes (n=3455) | *P*-value |
| **Maternal CES-D Score** | 10.36 ± 6.16 | 14.65 ± 7.49 | <0.001 |
| **Maternal CES-D Score** |  |  | <0.001 |
| <16 | 11971 (87.64%) | 2381 (68.91%) |  |
| ≥16 | 1689 (12.36%) | 1074 (31.09%) |  |
| **Maternal age (y)** |  |  | <0.001 |
| ≤30 | 2003 (14.92%) | 610 (18.19%) |  |
| >30 ≤40 | 10100 (75.25%) | 2471 (73.70%) |  |
| >40 | 1319 (9.83%) | 272 (8.11%) |  |
| **Child age (y)** |  |  | 0.202 |
| ≤4 | 3152 (23.74%) | 740 (22.32%) |  |
| >4 ≤5 | 4391 (33.08%) | 1104 (33.30%) |  |
| >5 | 5732 (43.18%) | 1471 (44.37%) |  |
| **Child Gender** |  |  | 0.003 |
| Boy | 7028 (51.45%) | 1680 (48.63%) |  |
| Girl | 6632 (48.55%) | 1775 (51.37%) |  |
| **Maternal educational level** |  |  | <0.001 |
| Below high School | 3570 (26.13%) | 1306 (37.80%) |  |
| High School | 6075 (44.47%) | 1434 (41.51%) |  |
| Above high School | 4015 (29.39%) | 715 (20.69%) |  |
| **Maternal employment status** |  |  | <0.001 |
| employed | 9378 (68.65%) | 2031 (58.78%) |  |
| unemployed | 4282 (31.35%) | 1424 (41.22%) |  |
| **Annual family income, in thousands, ¥** |  |  | <0.001 |
| <¥100 | 11032 (80.76%) | 3038 (87.93%) |  |
| ≥¥100 | 2628 (19.24%) | 417 (12.07%) |  |
| **Maternal marital status** |  |  | <0.001 |
| Married | 13375 (97.91%) | 3336 (96.56%) |  |
| non-married | 285 (2.09%) | 119 (3.44%) |  |

Data are expressed as number (percentage).

Note: ¥, yuan renminbi (to convert to US dollar, multiply by 7.30).

**Table S3. Supplementary data for Table 2**

|  | **Externalizing problems** | |  |
| --- | --- | --- | --- |
|  | No (n=13020) | Yes (n=4095) | *P*-value |
| **Maternal CES-D Score** | 10.50 ± 6.33 | 13.53 ± 7.22 | <0.001 |
| **Maternal CES-D Score** |  |  | <0.001 |
| <16 | 11313 (86.89%) | 3039 (74.21%) |  |
| ≥16 | 1707 (13.11%) | 1056 (25.79%) |  |
| **Maternal age (y)** |  |  | 0.005 |
| ≤30 | 1933 (15.11%) | 680 (17.06%) |  |
| >30 ≤40 | 9614 (75.17%) | 2957 (74.18%) |  |
| >40 | 1242 (9.71%) | 349 (8.76%) |  |
| **Child age (y)** |  |  | <0.001 |
| ≤4 | 2871 (22.69%) | 1021 (25.93%) |  |
| >4 ≤5 | 4210 (33.27%) | 1285 (32.64%) |  |
| >5 | 5572 (44.04%) | 1631 (41.43%) |  |
| **Child Gender** |  |  | 0.006 |
| Boy | 6548 (50.29%) | 2160 (52.75%) |  |
| Girl | 6472 (49.71%) | 1935 (47.25%) |  |
| **Maternal educational level** |  |  | <0.001 |
| Below high School | 3601 (27.66%) | 1275 (31.14%) |  |
| High School | 5771 (44.32%) | 1738 (42.44%) |  |
| Above high School | 3648 (28.02%) | 1082 (26.42%) |  |
| **Maternal employment status** |  |  | 0.274 |
| employed | 8708 (66.88%) | 2701 (65.96%) |  |
| unemployed | 4312 (33.12%) | 1394 (34.04%) |  |
| **Annual family income, in thousands, ¥** |  |  | <0.001 |
| <¥100 | 10620 (81.57%) | 3450 (84.25%) |  |
| ≥¥100 | 2400 (18.43%) | 645 (15.75%) |  |
| **Maternal marital status** |  |  | 0.145 |
| Married | 12725 (97.73%) | 3986 (97.34%) |  |
| non-married | 295 (2.27%) | 109 (2.66%) |  |

Data are expressed as number (percentage).

Note: ¥, yuan renminbi (to convert to US dollar, multiply by 7.30).

**Table S4. Supplementary data for Table 2**

|  | **Prosocial behavior problems** | |  |
| --- | --- | --- | --- |
|  | No (n=8853) | Yes (n=8262) | *P*-value |
| **Maternal CES-D Score** | 10.17 ± 6.52 | 12.36 ± 6.66 | <0.001 |
| **Maternal CES-D Score** |  |  | <0.001 |
| <16 | 7735 (87.37%) | 6617 (80.09%) |  |
| ≥16 | 1118 (12.63%) | 1645 (19.91%) |  |
| **Maternal age (y)** |  |  | <0.001 |
| ≤30 | 1281 (14.69%) | 1332 (16.53%) |  |
| >30 ≤40 | 6509 (74.65%) | 6062 (75.25%) |  |
| >40 | 929 (10.65%) | 662 (8.22%) |  |
| **Child age (y)** |  |  | <0.001 |
| ≤4 | 1713 (19.86%) | 2179 (27.35%) |  |
| >4 ≤5 | 2868 (33.26%) | 2627 (32.98%) |  |
| >5 | 4043 (46.88%) | 3160 (39.67%) |  |
| **Child Gender** |  |  | <0.001 |
| Boy | 4297 (48.54%) | 4411 (53.39%) |  |
| Girl | 4556 (51.46%) | 3851 (46.61%) |  |
| **Maternal educational level** |  |  | <0.001 |
| Below high School | 2334 (26.36%) | 2542 (30.77%) |  |
| High School | 3915 (44.22%) | 3594 (43.50%) |  |
| Above high School | 2604 (29.41%) | 2126 (25.73%) |  |
| **Maternal employment status** |  |  | <0.001 |
| employed | 6013 (67.92%) | 5396 (65.31%) |  |
| unemployed | 2840 (32.08%) | 2866 (34.69%) |  |
| **Annual family income, in thousands, ¥** |  |  | <0.001 |
| <¥100 | 7098 (80.18%) | 6972 (84.39%) |  |
| ≥¥100 | 1755 (19.82%) | 1290 (15.61%) |  |
| **Maternal marital status** |  |  | 0.055 |
| Married | 8625 (97.42%) | 8086 (97.87%) |  |
| non-married | 228 (2.58%) | 176 (2.13%) |  |

Data are expressed as number (percentage).

Note: ¥, yuan renminbi (to convert to US dollar, multiply by 7.30).

**Table S5. Sensitivity Analysis: Threshold effect analysis with dummy variable adjustment for missing data (N=17,115) compared to primary analysis (N=16,258, Table 3)**

|  | **Total difficulties score** | **Internalizing problems** | **Externalizing problems** | **Prosocial behavior** **problems** |
| --- | --- | --- | --- | --- |
|  | **OR (95%CI) *P* value** | **OR (95%CI) *P* value** | **OR (95%CI) *P* value** | **OR (95%CI) *P* value** |
| **Model I** |  |  |  |  |
| One line effect | 1.10 (1.10, 1.11) <0.0001 | 1.09 (1.08, 1.09) <0.0001 | 1.07 (1.06, 1.07) <0.0001 | 1.05 (1.05, 1.06) <0.0001 |
| **Model II** |  |  |  |  |
| Turning point (K) | 17 | 17 | 21 | 15 |
| CES-D < K | 1.20 (1.18, 1.21) <0.0001 | 1.16 (1.14, 1.17) <0.0001 | 1.10 (1.09, 1.11) <0.0001 | 1.10 (1.10, 1.11) <0.0001 |
| CES-D > K | 1.02 (1.01, 1.03) <0.0001 | 1.02 (1.01, 1.03) 0.0002 | 0.99 (0.98, 1.01) 0.2718 | 1.00 (0.99, 1.01) 0.7625 |
| *P* value for LRT test* | <0.001 | <0.001 | <0.001 | <0.001 |
| 95% CI for turning point | 15, 18 | 15, 18 | 16, 23 | 14, 17 |

Note: This sensitivity analysis assessed the robustness of threshold effects to missing data by including all eligible participants (N=17,115) using dummy variable adjustment for missing covariates (maternal age: n=340; child age: n=525), compared to the primary complete-case analysis (N=16,258, Table 3). Results demonstrated high consistency between approaches, with identical or nearly identical threshold values, overlapping 95% confidence intervals, and minimal differences in odds ratios (<1% variation), confirming the robustness of findings to missing data handling. Data are presented as OR (95% CI) P value. Model I: linear analysis assuming constant association across CES-D scores. Model II: non-linear analysis identifying threshold effects. All models adjusted for maternal age (years), child age (years), child gender, maternal education level, maternal employment status, maternal marital status, and annual family income level.

Abbreviations: CI, confidence interval; OR, odds ratio; LRT, logarithm likelihood ratio test.

**P* < 0.05 for LRT indicates that Model II fits significantly better than Model I.

**Table S6. Generalized Additive Mixed Model results accounting for kindergarten-level clustering** **(N=16,258)**

| **Outcome** | **Smooth Term** | | | **Fixed Effect** | | **Random Effect** | |
| --- | --- | --- | --- | --- | --- | --- | --- |
|  | **edf** | **F** | ***P*** | **β(95%CI)** | ***P*** | **ICC** | **DE** |
| Total Difficulties score | 5.13 | 213.36 | <0.001 | 3.60 (2.54-4.67) | <0.001 | 0.000 | 1.00 |
| Internalizing Problems | 5.57 | 162.86 | <0.001 | 2.80 (1.71-3.89) | <0.001 | 0.000 | 1.00 |
| Externalizing Problems | 4.64 | 137.06 | <0.001 | 2.06 (1.07-3.06) | <0.001 | 0.000 | 1.00 |
| Prosocial Behavior Problems | 4.77 | 120.79 | <0.001 | 0.65 (-0.32-1.62) | 0.187 | 0.000 | 1.00 |

Note: N=16,258 children nested within 189 kindergartens. edf = effective degrees of freedom for the smooth term s(CES-D); F = F-statistic for the smooth term; β = fixed effect coefficient for the parametric component of the smooth term; CI = confidence interval; ICC = intraclass correlation coefficient; DE = design effect. All models were fitted using Generalized Additive Mixed Model (GAMM) with kindergarten as a random intercept, adjusting for maternal age, child age, child gender, maternal education level, maternal employment status, maternal marital status, and annual family income level. Random intercept standard deviations were <0.001 for total difficulties score, internalizing problems, and prosocial behavior problems, and 0.0027 for externalizing problems. ICC was calculated as σ²_random / (σ²_random + π²/3), where π²/3 ≈ 3.29 for binomial models with logit link. Design effect was calculated as 1 + (n̄ - 1) × ICC, where n̄ = 86 is the average cluster size. The smooth term significance (indicated by edf and F-statistic) demonstrates the overall nonlinear association between MDS and child mental health outcomes, while β represents the parametric component of the smooth function.

**
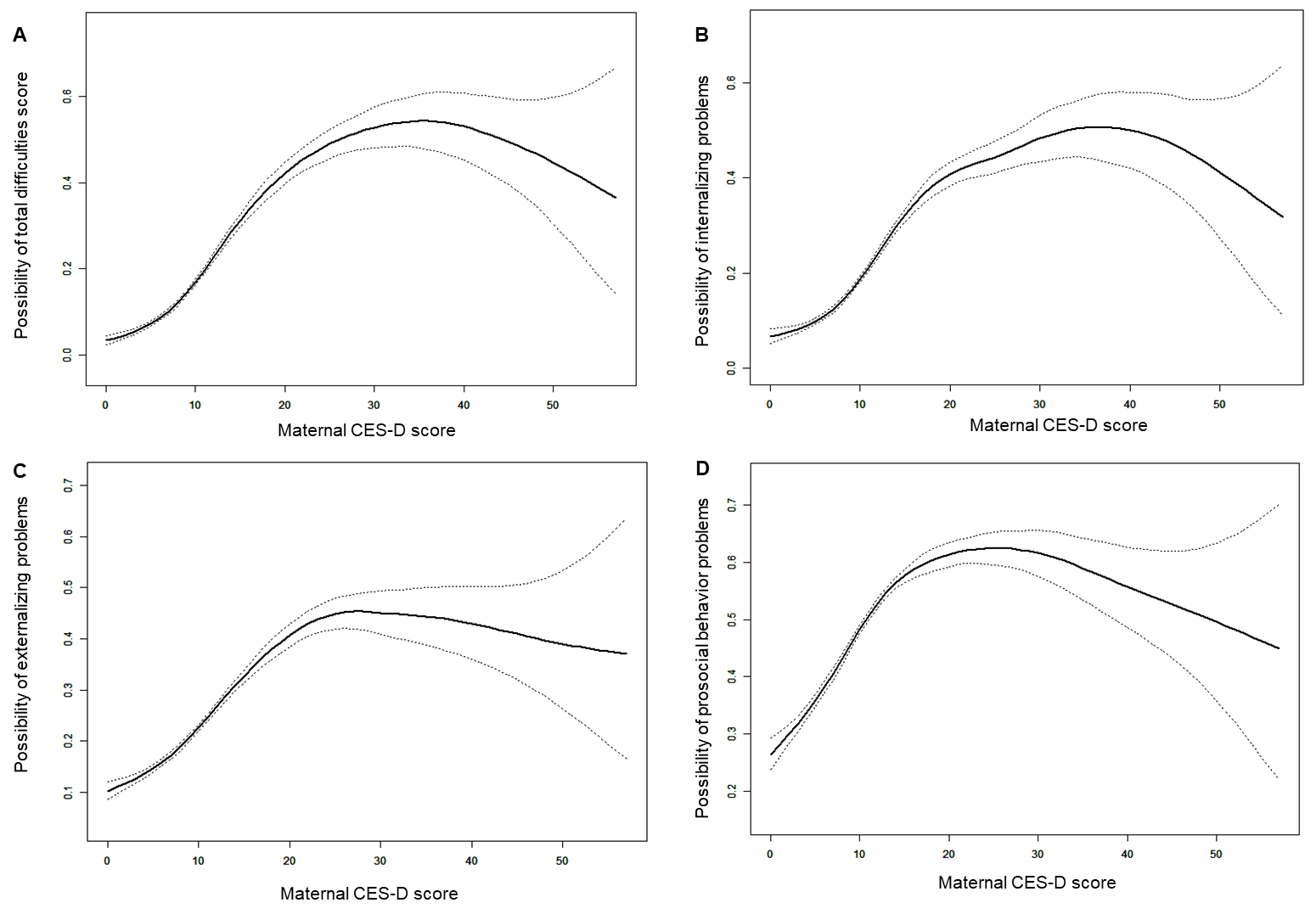
**

**Figure S1. Smooth terms for the nonlinear associations between MDS and children's mental health outcomes.** Solid lines represent the estimated smooth functions, and dashed lines represent 95% confidence intervals. Effective degrees of freedom (edf) and p-values are shown for each outcome. The nonlinear patterns remained consistent with primary GAM analyses, confirming robustness to kindergarten-level clustering.

**Table S7. Sensitivity Analysis: Threshold effect analysis using continuous SDQ scores (N=17,115)**

|  | **Total difficulties score** | **Internalizing problems** | **Prosocial behavior** |
| --- | --- | --- | --- |
|  | **β (95%CI) *P* value** | **β (95%CI) *P* value** | **β (95%CI) *P* value** |
| **Model I** |  |  |  |
| One line effect | 0.19 (0.18, 0.20) <0.0001 | 0.08 (0.07, 0.08) <0.0001 | -0.06 (-0.07, -0.06) <0.0001 |
| **Model II** |  |  |  |
| Turning point (K) | 23 | 23 | 14 |
| CES-D < K | 0.24 (0.22, 0.25) <0.0001 | 0.10 (0.09, 0.10) <0.0001 | -0.13 (-0.13, -0.12) <0.0001 |
| CES-D > K | 0.03 (0.00, 0.06) 0.0437 | 0.01 (-0.01, 0.02) 0.3243 | -0.01 (-0.02, -0.00) 0.0126 |
| *P* value for LRT test* | <0.001 | <0.001 | <0.001 |
| 95% CI for turning point | 21, 23 | 19, 23 | 13, 15 |

|  | **Hyperactivity** | **Conduct problems** |
| --- | --- | --- |
|  | **β (95%CI) *P* value** | **β (95%CI) *P* value** |
| **Model I** |  |  |
| One line effect | 0.06 (0.06, 0.07) <0.0001 | 0.05 (0.04, 0.05) <0.0001 |
| **Model II** |  |  |
| Turning point (K) | 17 | 23 |
| CES-D < K | 0.10 (0.09, 0.10) <0.0001 | 0.06 (0.05, 0.06) <0.0001 |
| CES-D > K | 0.01 (0.01, 0.02) 0.0005 | -0.00 (-0.01, 0.01) 0.3984 |
| *P* value for LRT test* | <0.001 | <0.001 |
| 95% CI for turning point | 15, 23 | 21, 23 |

This supplementary analysis examined threshold effects using continuous SDQ scores to assess the robustness of findings from the primary dichotomized analyses (Table 3). For externalizing problems (analyzed as a composite in Table 3), the two constituent subscales-hyperactivity and conduct problems—were analyzed separately to provide more granular insights. Results demonstrate consistent nonlinear patterns across operationalizations. Identified thresholds showed reasonable consistency: total difficulties (23 vs 17), internalizing problems (23 vs 17), and prosocial behavior (14 vs 14-15 in Table 3). Notably, hyperactivity showed a lower threshold (17) compared to conduct problems (23), with the composite externalizing threshold (21 in Table 3) falling between them. The consistent pattern of stronger associations below thresholds and attenuated effects above them confirms that threshold effects reflect substantive nonlinear relationships rather than artifacts of dichotomization. Model I presents linear associations; Model II identifies optimal threshold points using two-piecewise linear regression. All models adjusted for maternal age, child age, child gender, maternal education, employment status, marital status, and family income.

Note: β represents the change in SDQ score per 1-point increase in maternal CES-D score. CI, confidence interval; LRT, logarithm likelihood ratio test. **P* < 0.05 indicates Model II fits significantly better than Model I.


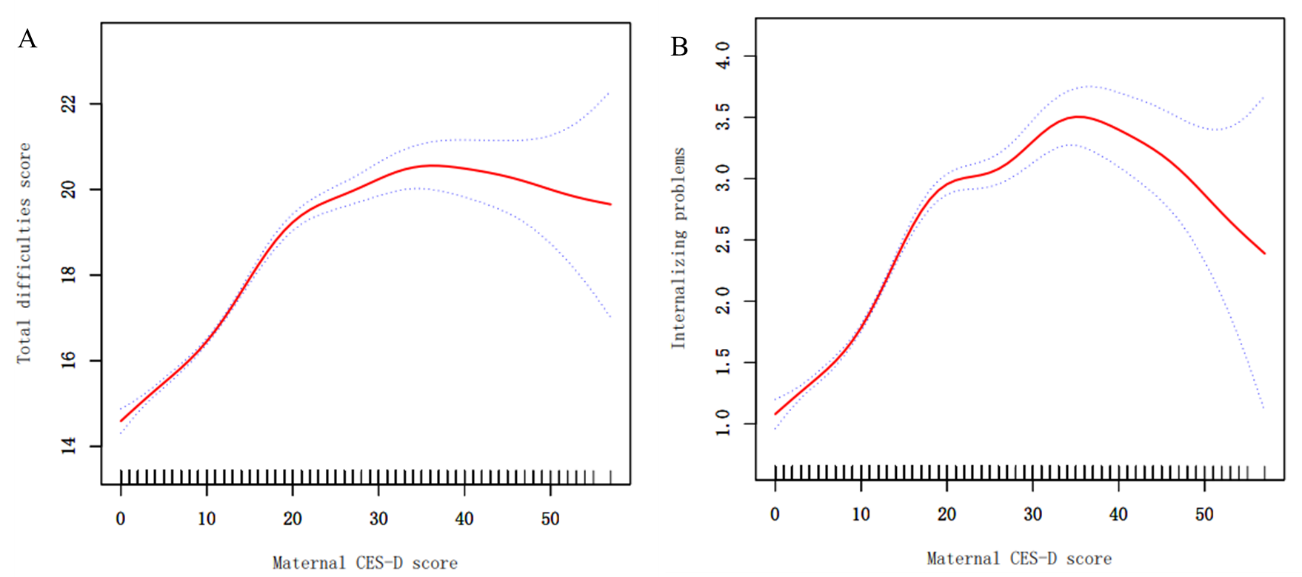


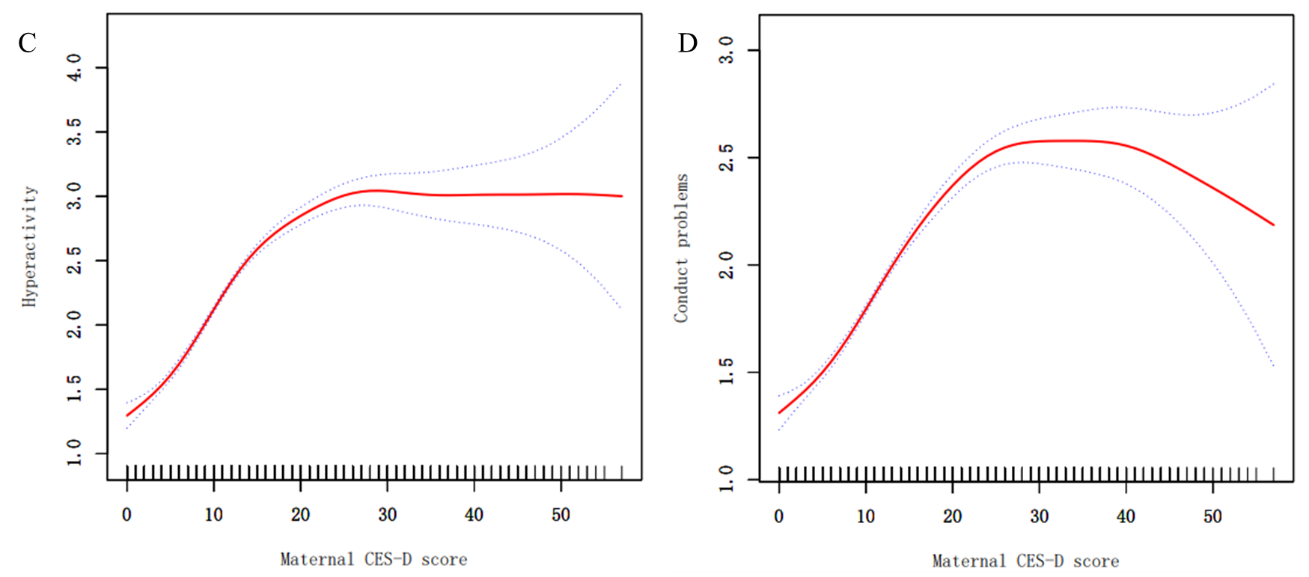


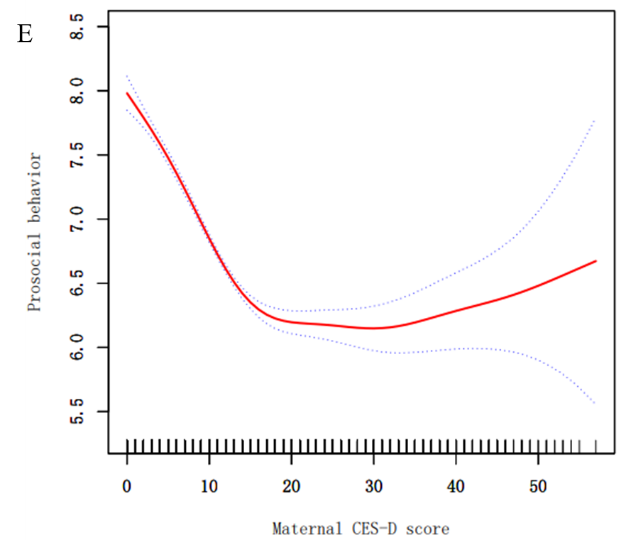


**Figure S2. Sensitivity analysis: Nonlinear associations between maternal depressive symptoms and continuous SDQ scores using generalized additive models (N=16,258).** Panel A shows the association between maternal CES-D score and total difficulties score; Panel B shows internalizing problems; Panel C shows hyperactivity; Panel D shows conduct problems; Panel E shows prosocial behavior score. The solid red line represents the smoothed curve fit between variables. Blue dotted bands represent the 95% confidence interval from the fit. These analyses using continuous SDQ scores demonstrate nonlinear patterns consistent with primary analyses using dichotomized outcomes (Figure 1), confirming the robustness of threshold effects. Notably, the direction of association for prosocial behavior (Panel E, decreasing scores indicating worsening prosocial skills) is conceptually consistent with the primary analysis (Figure 1D, increasing probability of prosocial behavior problems), as lower prosocial behavior scores indicate greater deficits. For externalizing problems, hyperactivity and conduct problems were analyzed separately to provide more granular insights into the constituent dimensions of the composite externalizing variable used in primary analyses.

Abbreviations: CES-D, Center for Epidemiologic Studies Depression Scale; SDQ, Strengths and Difficulties Questionnaire.

Note: Prosocial behavior scores range from 0-10, with lower scores indicating greater deficits (score <6 indicates prosocial behavior problems).
